# Prolonged continuous intravenous infusion of the dipeptide L-alanine- L-glutamine significantly increases plasma glutamine and alanine without elevating brain glutamate in patients with severe traumatic brain injury

**DOI:** 10.1186/cc13962

**Published:** 2014-07-02

**Authors:** Mirjam Nägeli, Mario Fasshauer, Jutta Sommerfeld, Angela Fendel, Giovanna Brandi, John F Stover

**Affiliations:** 1 Surgical Intensive Care Medicine, University Hospital Zuerich, Raemistrasse 100, Zuerich 8091, Switzerland

## Abstract

**Introduction:**

Low plasma glutamine levels are associated with worse clinical outcome. Intravenous glutamine infusion dose- dependently increases plasma glutamine levels, thereby correcting hypoglutaminemia. Glutamine may be transformed to glutamate which might limit its application at a higher dose in patients with severe traumatic brain injury (TBI). To date, the optimal glutamine dose required to normalize plasma glutamine levels without increasing plasma and cerebral glutamate has not yet been defined.

**Methods:**

Changes in plasma and cerebral glutamine, alanine, and glutamate as well as indirect signs of metabolic impairment reflected by increased intracranial pressure (ICP), lactate, lactate-to-pyruvate ratio, electroencephalogram (EEG) activity were determined before, during, and after continuous intravenous infusion of 0.75 g L-alanine-L-glutamine which was given either for 24 hours (group 1, *n* = 6) or 5 days (group 2, *n* = 6) in addition to regular enteral nutrition. Lab values including nitrogen balance, urea and ammonia were determined daily.

**Results:**

Continuous L-alanine-L-glutamine infusion significantly increased plasma and cerebral glutamine as well as alanine levels, being mostly sustained during the 5 day infusion phase (plasma glutamine: from 295 ± 62 to 500 ± 145 μmol/ l; brain glutamine: from 183 ± 188 to 549 ± 120 μmol/ l; plasma alanine: from 327 ± 91 to 622 ± 182 μmol/ l; brain alanine: from 48 ± 55 to 89 ± 129 μmol/ l; p < 0.05, ANOVA, post hoc Dunn’s test).

Plasma glutamate remained unchanged and cerebral glutamate was decreased without any signs of cerebral impairment. Urea and ammonia were significantly increased within normal limits without signs of organ dysfunction (urea: from 2.7 ± 1.6 to 5.5 ± 1.5 mmol/ l; ammonia: from 12 ± 6.3 to 26 ± 8.3 μmol/ l; p < 0.05, ANOVA, post hoc Dunn’s test).

**Conclusions:**

High dose L-alanine-L-glutamine infusion (0.75 g/ kg/ d up to 5 days) increased plasma and brain glutamine and alanine levels. This was not associated with elevated glutamate or signs of potential glutamate-mediated cerebral injury. The increased nitrogen load should be considered in patients with renal and hepatic dysfunction.

**Trial registration:**

Clinicaltrials.gov NCT02130674. Registered 5 April 2014

## Introduction

Low plasma glutamine is associated with increased mortality and aggravated functional impairment in critically ill patients [1]. Thus, adequate substitution of the conditionally essential amino acid glutamine in critically ill patients is strongly recommended by international societies with a grade-A level of evidence [2,3]. Glutamine essentially supports protein synthesis, mitosis, muscle growth, immune function, formation of the antioxidant glutathione, and prevents apoptosis [4]. In addition, glutamine as a gluconeogenic and lipogenic precursor, increases cellular energetic reserves, stimulates insulin release, and ameliorates glucose metabolism under conditions of insulin resistance in critically ill patients [4,5]. Glutamine administration reduces infection-related increased morbidity [5-9], decreases mortality during the intensive care phase [10,11], shortens length of hospitalization [12,13], thereby substantially reducing hospital costs [12-14]. The optimal dose, however, still remains to be determined as concluded in the SIGNET trial [15].

Plasma glutamine levels can be increased by enteral as well as intravenous administration of glutamine [16]. However, metabolic conversion of glutamine increases plasma glutamate levels especially during enteral glutamine administration [16]. In patients suffering from a damaged blood-brain barrier as encountered following severe traumatic brain injury (TBI) an increase in plasma glutamate is feared to induce additional brain damage due to its excitotoxic and edema-aggravating potential [17-19]. As shown by Berg and colleagues [20], intravenous infusion of the dipeptide L-alanine-L-glutamine administering 0.34 g glutamine/kg limited to a 20-hour infusion period increased plasma glutamine levels by 30% without elevating arterial plasma or cerebral glutamate and without signs of cerebral glutamate uptake in patients with severe head trauma (Glasgow coma scale (GCS) score ≤8) [21]. In addition, these results suggest that a glutamine dose exceeding 0.34 g/kg body weight might be required to normalize decreased plasma glutamine levels in individual patients. However, before a higher glutamine dose can be considered safe in patients suffering from severe TBI, a glutamine-dependent increase in cerebral glutamate and signs of cerebral impairment must be excluded during prolonged infusion of a higher glutamine dose. For this, we prospectively investigated the effects of continuous intravenous infusion of 0.5 g glutamine/kg/d (corresponding to 0.75 g/ kg/d Dipeptiven® = L-alanine-L-glutamine; 82 mg/ml L-alanine, 134.6 mg/ ml L-glutamine; Fresenius Kabi, Switzerland) for 24 hours (study 1, six patients) and 5 days (study 2, six patients). Infusion-related analysis included changes in arterial, jugular venous, and cerebral glutamine, alanine, and glutamate levels as well as alterations in brain glucose, lactate, and pyruvate. In addition, changes in intracranial pressure (ICP), cerebral perfusion pressure (CPP), brain tissue oxygenation (ptiO_2_), and electroencephalographic activity assessed by bispectral index electroencephalogram (BIS EEG) technology were continuously recorded in patients with traumatic brain injury (TBI) subjected to pharmacologic coma, using fentanyl and midazolam.

## Materials and methods

In study 1, a total of six patients were included to investigate the effects of 0.5 g glutamine/kg/d (Dipeptiven® = L-alanine-L-glutamine: 82 mg/100 ml L-alanine and 134.6 mg/100 ml L-glutamine) continuously infused for 24 hours followed by a 24-hour observation period. In study 2, a total of six patients were included to investigate the effects of 0.5 g glutamine/kg/d (Dipeptiven® = L-alanine-L-glutamine; 82 mg L-alanine, 134.6 mg L-glutamine) continuously infused for 5 days followed by a 48-hour observation period. The study protocol was approved by the local ethics committee (*Kantonale Ethikkommission* Zürich) and written informed consent was obtained by the relatives of the patients.

### Inclusion criteria

Patients suffering from severe TBI reflected by abnormal neurologic status and pathologic neuroradiologic findings were considered eligible when requiring pharmacologic coma. Patients were only included if the different catheters (arterial and central venous lines, neuromonitoring probes; *vide infra*) and triluminal jejunal feeding tube were in place and functional for at least 12 hours before infusing L-alanine-L-glutamine.

Patients who were anticipated to die within 48 hours with abdominal injury, mass transfusion, renal or hepatic impairment, requiring barbiturates to treat intracranial hypertension, or receiving parenteral nutrition, and having body weight below 50 kg or exceeding 100 kg were not included.

### Study protocol (study 1 and study 2)

**Figure 1:**
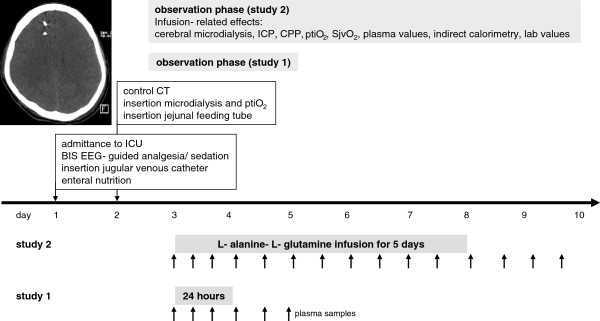
**Study protocol investigating the effects of continuously infusing L-alanine-L- glutamine (Dipeptiven®** = **L-alanine-L-glutamine; 82 mg L-alanine, 134.6 mg L-glutamine; 0.5 g glutamine/kg) for 24 hours (study 1) or 5 days (study 2) in six patients each suffering from severe traumatic brain injury.** The representative computed tomography (CT) scan shows the position of the microdialysis catheter and intracranial pressure probe. ICP, intracranial pressure; CPP, cerebral perfusion pressure; ptiO_2_, brain tissue oxygen; SjvO_2_, jugular venous oxygen saturation; BIS EEG, bispectral index electroencephalogram.

Following admission to the trauma surgical ICU, all patients were treated according to a standardized protocol as published previously [22,23]. For this, a BIS EEG sensor was applied to guide analgesia and sedation (Figure 1); a jugular venous catheter was inserted within the first hour to guide ventilation and adjust analgesia/sedation and CPP; after approximately 24 hours a control cranial computed tomography (CT) scan was performed to determine the site of insertion of the microdialysis (CMA 70, 10 mm membrane, CMA/ Microdialysis, Dallvägen 10, Solna Sweden) and ptiO_2_ catheters (LICOX® IMC Oxygen Catheter Micro Probe, Integra NeuroSciences, Plainsboro, NJ, USA) to avoid penetrating frontal contusions, as these would not yet be visible on the first CT and tend to develop during the first 24 hours. Microdialysis, ptiO_2_ and temperature (LICOX® IMC Temperature Micro Probe; Integra NeuroSciences) probes were inserted using a triluminal bolt system (LICOX® IMC Bolt System, Triple Lumen; Integra NeuroSciences). The patients were fed enterally via a gastric feeding tube beginning on the day of admission to the ICU, using Fresubin® Energy fibre (Fresenius Kabi AG, Switzerland), containing 5.6 g protein/100 ml with 0.62 g glutamine/100 ml and 0.2 g alanine/100 ml with 1.5 kcal/ml. On the second post-traumatic day a jejunal feeding tube was positioned endoscopically by the gastroenterologists and enteral nutrition with Fresubin® Energy fibre was continued. None of the patients received parenteral nutrition in addition to the enteral nutrition. According to our standardized nutritional protocol, enteral nutrition was begun within 12 hours after admission to the ICU, started at 10 ml/h and increased by 10 ml/h in 6-hour intervals until reaching the measured caloric requirement determined by indirect calorimetry (Deltatrac™ II; Datex Instruments, Helsinki, Finland). On the third post-traumatic day, patients in study 1 received L-alanine-L-glutamine at 0.5 g glutamine/kg/d by continuous intravenous infusion for 24 hours, followed by a 24-hour observation period. In study 2, aimed at excluding an accumulation effect, L-alanine-L-glutamine was continuously infused intravenously at 0.5 g glutamine/kg/d starting on the third posttraumatic day lasting for five days, followed by an additional 48-hour observation phase.

Cerebral microdialysis samples were measured hourly to monitor metabolic alterations reflected by changes in glucose, lactate, pyruvate, and glutamate. The remaining volume was used to analyze changes in alanine, glutamine, and glutamate by high performance liquid chromatography (HPLC). In study 1, arterial and jugular venous plasma samples were drawn at predefined time points before, during (1, 4, 12, and 23 hours during the infusion period), and after the infusion period (4, 12, and 23 hours after the infusion). Indirect calorimetry was performed before, during (8.0 and 23.5 hours), and after the infusion period (6.0 and 23.5 hours).

In study 2, plasma arterial and jugular venous samples were drawn at predefined time points before, during (1, 4, 12, 24, 36, 48, 60, 72, 84, 96, 108, and 120 hours), and after the infusion period (4, 12, 23, and 48 hours after the infusion). Indirect calorimetry was performed before, during (12-hour intervals), and after the infusion period (12-hour intervals). The results obtained during these investigations were blinded to the clinicians and, thus, did not influence clinical decision-making.

In both studies, predefined signs of glutamate-mediated cerebral injury were actively sought by the study team (JFS, MF, JS) with the aim of stopping the L-alanine-L-glutamine infusion in case of cerebral metabolic impairment reflected by elevated cerebral glutamate, increased lactate and lactate/pyruvate ratio, decreased ptiO_2_, elevated ICP, increased BIS EEG activity, and escalation in therapeutic interventions, respectively. Here, the following threshold values were defined a priori: a 2-fold increase in plasma or cerebral glutamate associated with a 2-fold increase in cerebral lactate or lactate/pyruvate ratio, or ICP >20 mmHg, or SjvO_2_ < 60%, or arterial-jugular venous oxygen difference < -0.2, or BIS EEG >40, or a more aggressive treatment approach.

### Investigated parameters

Bedside analysis of glutamate was performed enzymatically using the bedside ISCUSFlex Microdialysis Analyzer (CMA/Microdialysis, Dalvägen 10, Solna, Sweden) to guide possible termination of L-alanine L-glutamine infusion. In addition, metabolic parameters, that is, glucose, lactate, pyruvate, and calculated lactate/pyruvate ratio were also determined enzymatically. Glutamine, glutamate, and alanine were determined in arterial and jugular venous plasma and cerebral microdialysis by HPLC.

ICP, CPP, BIS EEG were recorded continuously using the LabPilot™ software (CMA/Microdialysis, Dalvägen 10, Solna, Sweden). ptiO_2_ (LICOX® IMC Oxygen Catheter Micro Probe) and brain temperature (LICOX® IMC Temperature Micro Probe) (Integra NeuroSciences) were recorded continuously using the LabPilot™ software (CMA/ Microdialysis, Dalvägen 10, Solna, Sweden). Indirect calorimetry was performed using the Deltatrac™ II (Datex Intrsuments, Helsinki, Finland), which also allowed us to determine the respiratory quotient (RQ).

Routine laboratory values were determined once daily assessing changes in lymphocytes, creatinine, urea, and ammonia. Nitrogen balance was calculated by subtracting urinary nitrogen determined in urine collected over 24 hours from the daily nitrogen intake (enteral nutrition plus L-alanine-L-glutamine infusion); possible nitrogen loss via, for example, skin and stool were not considered.

### HPLC analysis of amino acids

Plasma samples were drawn using commercially available pre-heparinized syringes (*safe* PICO Aspirator, Radiometer, Copenhagen, Radiometer Medical ApS, Åkadevej 21, DK- 2700 Brønshøj, Denmark). Following centrifugation at 4°C and 10,000 rpm for 10 minutes (HETTICH Zentrifugen Universal 30 F/ RF, HETTICH AG, 8806 Bäch, Switzerland) samples were deproteinized with acetonitrile (plasma 1:7, microdialysate 1:3). Following centrifugation the supernatant was frozen at -80˚C until further analysis.

Following unthawing, samples were vortexed and spiked with the internal standard norvaline (NVA), centrifuged, and applied to a vacuum manifold system (Multi-well Filter Plate Vacuum Manifold and Accessories for 96-well Filter Plates, Pall Corporation, USA, Port Washington, NY 11050) using a vacuum of 77.5 mmHg for 15 seconds. Thereafter the samples were transferred to a cooled autosampler maintained at 4˚C. Before automated injection, samples were incubated with ortho-phthaldialdehyde (OPA) (1:1) for 1 minute. After injection, a stepped gradient at a flow rate of 1.5 ml/min was applied (buffer B: 0, 0, 30, 50, 100, 0, 0% at 0, 0.8, 12, 16, 16.2, and 17 minutes) with a total run time of 18 minutes. HPLC was linked to a fluorescence detector (Agilent HPLC 1100 Serie, Agilent Technologies AG, Basel, Switzerland) set at 340 nm (excitation) and 450 nm (emission wavelength). Stationary phase was a ZORBAX Eclipse AAA column (4.6 × 75 mm, 3.5-μm particle size) (Agilent Technologies AG). The following mobile phases were used: buffer A: 5.5 g sodium dihydrogen phosphate anhydrous (H_2_NaO_4_P) + 1 l Aqua HPLC (purified, filtered distilled water) and buffer B: 450 ml acetonitrile + 450 ml methanol + 100 ml Aqua HPLC (sodium dihydrogen phosphate anhydrous, acetonitrile, and methanol were purchased from Sigma-Aldrich Chemie GmbH, Buchs, Switzerland).

A standard mixture of the amino acids (AA) analyzed as an external standard was also spiked using the same internal standard (NVA) as in the plasma and microdialysis samples. OPA, AA and NVA standards were purchased from Agilent Technologies AG, Basel, Switzerland.

### Standardized intensive care treatment following severe traumatic brain injury

All patients were treated on our ICU according to a standardized protocol as published previously [22,23] with integrated extended neuromonitoring [24]. Routine treatment and decision-making were not influenced by the present investigations. Continuously infused midazolam (Dormicum®) and fentanyl (Sintenyl®) were tapered according to ICP and BIS EEG values, maintaining ICP values <15 mmHg and BIS EEG values between 20 and 40. Volume and norepinephrine were adjusted to maintain CPP values between 60 and 90 mm Hg. Transcranial color-coded Duplex sonography was performed daily to guide CPP (including volume management) and ventilator settings (paCO_2_ levels) according to changes in flow velocity determined in the middle cerebral artery [25]. Ventilation, oxygenation, CPP, and blood transfusions were guided by maintaining ptiO_2_ > 15 mmHg.

### Statistical analysis

Results of the different parameters assessed at the predefined time points in the individual patients were presented as box plots. Cerebral glutamine, alanine, and glutamate values were determined by HPLC in hourly collected samples pooled in 4-hour blocks resulting in six values to calculate a median per day per patient; the box plots represent six daily median values for the six patients in study 1 and six patients in study 2. In study 2, the daily medians are compiled in one box plot after having excluded significant differences during the prolonged infusion phase. Statistical differences in changes before, during and after L-alanine-L-glutamine infusion and between the study groups were evaluated by one-way analysis of variance followed by Dunn’s multiple comparison test. Differences were rated significant at *P* <0.05.

## Results

### Demographic data

The investigated twelve patients in the two separate studies were comparable presenting with mixed lesions, predominantly consisting of contusions combined with hemispheric edema. In study 1, the two female and four male patients suffered from severe TBI (median Abbreviated Injury Score (AIS) 4, range 3 to 5) with a median age of 30 (range 19 to 45) and a median body mass index of 21 kg/m^2^ (range 19 to 23) required prolonged analgesia and sedation with extended neuromonitoring for a median of 13 days (range 7 to 34 days). Microdialysis and ptiO_2_/ temperature probes were inserted in the more severely injured hemisphere with two in the left frontal and four in the right frontal lobe, respectively. Patients survived with a median extended Glasgow Outcome Score (GOS) of 6 (range 5 to 8) at 12 months following posttraumatic neurorehabilitation.

In study 2, the two female and four male patients suffered from severe TBI (median AIS 4, range 3 to 5) with a median age of 28 years (range 17 to 42) and a median body mass index of 23 kg/m^2^ (range 21 to 26) required prolonged analgesia and sedation with extended neuromonitoring for a median of 14 days (range 9 to 37 days). Neuromonitoring probes were inserted in the more severely injured hemisphere with three in the left frontal and three in the right frontal lobe, respectively. Insertion of probes did not induce any additional tissue damage as revealed by subsequent CT imaging. Patients survived with a median extended Glasgow Outcome Score (GOS) of 6 (range 5 to 8) at 12 months following post-traumatic neurorehabilitation.

### Amount of infused L-alanine-L-glutamine and enteral nutrition

For the administration of 0.5 g glutamine/kg/d a median of 13 ml L-alanine-L-glutamine (Dipeptiven®) was infused per hour during the 24-hour (study 1) and 5-day (study 2) group, respectively (Table 1). This resulted in a cumulative median amount of 312 ml per day (range 216 to 360 ml) (study 1) and 1,560 ml for 5 days (range 1,080 to 1,800 ml) (study 2).

**Table 1:** Changes in enteral nutrition, neuromonitoring, caloric requirement, and laboratory values before, during, and after prolonged continuous L-alanine-L-glutamine infusion

	**Before infusion**	**During infusion**	**After infusion**
**Enteral nutrition and L-alanine-L-glutamine infusion**			
Enteral nutrition (ml/h)	42 ± 8, 30, 62	45 ± 11, 25, 60	47 ± 15, 30, 90
Calories (kcal/d)	1512 ± 288, 1,080, 2,232	1620 ± 396, 900, 2,160	1692 ± 540, 1,080, 3,240
L-alanine-L-glutamine (ml/h)	--	13 ± 2, 9, 15	--
**Neuromonitoring**			
ICP (mmHg)	11 ± 4, 4, 18	9 ± 5, 0, 40	9 ± 6, 0, 25
CPP (mmHg)	78 ± 6, 71, 90	79 ± 7, 60, 98	80 ± 8, 56, 110
Brain temperature (°C)	35.1 ± 0.2, 34.8, 35.4	35.4 ± 0.26, 34.6, 36	35.5 ± 0.5, 34.5, 37
ptiO_2_ (mmHg)	23 ± 11, 10, 40	29 ± 9, 15, 65	27 ± 8, 11, 57
BIS	33 ± 8, 20, 45	35 ± 12, 10, 94	42 ± 18, 10, 89*
Brain glucose (mM)	0.76 ± 0.99; 0.5, 3.5	1.1 ± 1.1; 0.6, 4.6	0.68 ± 1.0; 0.4, 6,5
Brain lactate (mM)	2.2 ± 3.5; 0.8, 11	3.8 ± 1.6; 0.5, 8.5	3.2 ± 1.7; 0.5, 9.3
Brain lactate-to-pyruvate ratio	14 ± 25; 2.4, 88	14 ± 23; 2, 136	9 ± 22; 3, 300
**Caloric requirement**			
Kcal	1,830 ± 193; 1,440, 1,940	1,945 ± 320; 1,480, 2,810	2,115 ± 353; 1,830, 2,830*
RQ	0.84 ± 0.07; 0.76, 0.94	0.84 ± 0.05; 0.76, 0.96	0.83 ± 0.07;0.72, 0.9
**Laboratory values**			
CRP	149 ± 79, 1.3, 203	109 ± 78; 25, 371	76 ± 69; 12, 269
Fibrinogen	4 ± 1.7; 1.7, 7.1	6.2 ± 1.5; 2.3, 8.5	6.5 ± 1.8; 2.2, 11.6
Albumin (g/L)	23 ± 4; 13, 29	22 ± 4; 14, 31	21 ± 5; 14, 33
Blood urea (mmol/L)	2.7 ± 1.6; 1.2, 7	5.5 ± 1.5; 1.6, 9.7*	5.1 ± 2.2; 2.2, 10.5
Ammonia (μmol/L)	12 ± 6.3; 6, 25	26 ± 8.3; 10, 51*	26 ± 8.4; 4, 34*
Creatinine (μmol/L)	68 ± 23; 53, 126	66 ± 16; 42, 122	68 ± 17; 45, 99
GOT (U/ml)	44 ± 61, 15, 206	42 ± 40; 13, 165	60 ± 53; 13, 191
GPT (U/ml)	20 ± 50; 11, 141	19 ± 64, 10, 172	18 ± 58, 12, 158
Urine urea (mmol/L)	74 ± 56; 40, 203	136 ± 85; 31, 398	117 ± 53; 36, 215
Nitrogen balance (g/d)	8 ± 3.6; 5.9, 10.7	10 ± 1.6; 6.9, 11	8.5 ± 3.3; 6, 16.5
Lymphocytes (10^-3^/μl)	0.9 ± 0.4; 0.37, 1.58	0.9 ± 0.3; 0.38, 1.59	1.0 ± 0.7; 0.72, 3.17
**Sedation**			
Midazolam (mg/h)	90 ± 20; 60, 110	90 ± 20; 60, 110	70 ± 20; 40, 90*

Nitrogen balance was calculated based on administered nitrogen from protein and amino acids minus nitrogen in urinary urea without including other potential losses via, for example, skin and stool. Results are shown as median ± standard deviation; range. *P <0.001 analysis of variance and post hoc Dunn’s test. ICP, CPP, BIS, ptiO_2_, RQ, respiratory quotient; CRP, C-reactive protein; GOT, aspartate aminotransferase; GPT, alanine aminotransferase.

Enteral nutrition was continuously applied without any interruptions, resulting in an overall median amount of 45 ml per hour before, during, and after the L-alanine-L-glutamine infusion phase (studies 1 and 2) (Table 1).

### Changes in plasma glutamine, alanine, and glutamate levels

**Figure 2:**
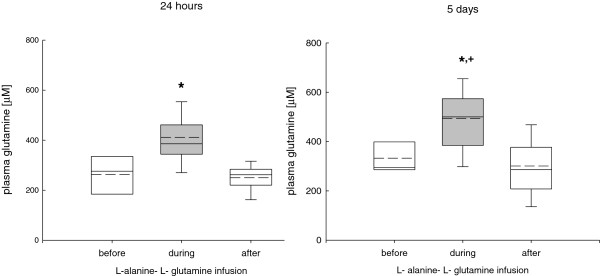
**Changes in plasma glutamine during the 24-hour and 5-day L-alanine-L-glutamine infusion phase.** During the infusion phase plasma glutamine was significantly and reversibly increased compared to baseline values (*P <0.001, analysis of variance, post hoc Dunn’s test) which was sustained during the 5-day compared to the 24-hour infusion phase (^+^*P* <0.001).

**Figure 3:**
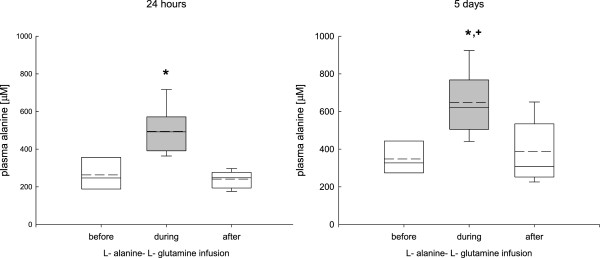
**Changes in plasma alanine during the 24-hour and 5-day L- alanine-L-glutamine infusion phase.** During the infusion phase plasma alanine was significantly increased compared to baseline values (*p <0.001, analysis of variance, post hoc Dunn’s test), which was mostly sustained during the 5-day infusion phase compared to the 24-hour infusion (^+^*P* <0.001). Although elevated plasma alanine levels reached baseline values following the 24-hour infusion phase, plasma alanine levels remained increased following the 5-day infusion phase.

Continuous infusion of L-alanine-L-glutamine significantly and reversibly increased plasma glutamine levels from 295 ± 62 μmol/L to 395 ± 175 μmol/L during the 24-hour phase and to 500 ± 145 μmol/L during the 5-day infusion phase, reaching the highest values during the 5-day infusion period (Figure 2).During the 24-hour and 5-day infusion phase plasma alanine was significantly and reversibly increased from 327 ± 91 μmol/L reaching highest values during the 5-day infusion period (622 ± 182 μmol/L) (Figure 3). Following the 24-hour infusion phase, plasma alanine returned to baseline levels whereas plasma alanine levels remained significantly increased following the 5-day infusion phase (Figure 3). Plasma glutamate levels remained unchanged during the entire study period (before infusion, 62 ± 18; during infusion, 54 ± 22; after infusion, 57 ± 19 μmol/L), irrespective of infusion duration.

Overall, there were no significant differences between arterial and jugular venous alanine, glutamine, and glutamate levels (data not shown).

### Changes in cerebral glutamine, alanine, and glutamate levels

**Figure 4:**
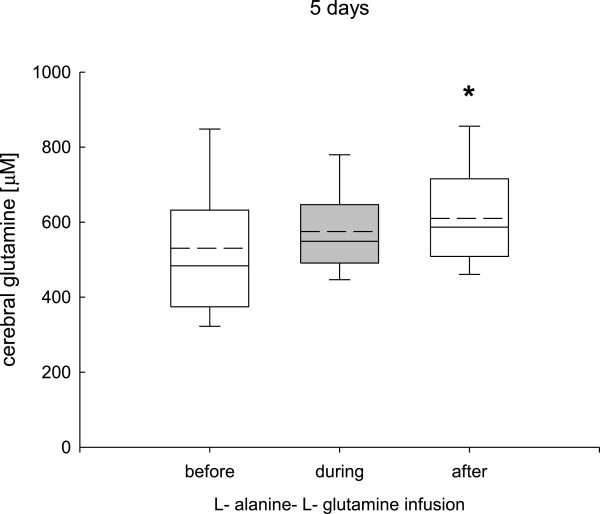
**Changes in cerebral glutamine determined by cerebral microdialysis during the 5-day L-alanine-L-glutamine infusion phase.** Cerebral glutamine remained unchanged during the infusion phase and showed a significant increase after the 5-day infusion period (*P <0.001, analysis of variance, post hoc Dunn’s test).

**Figure 5:**
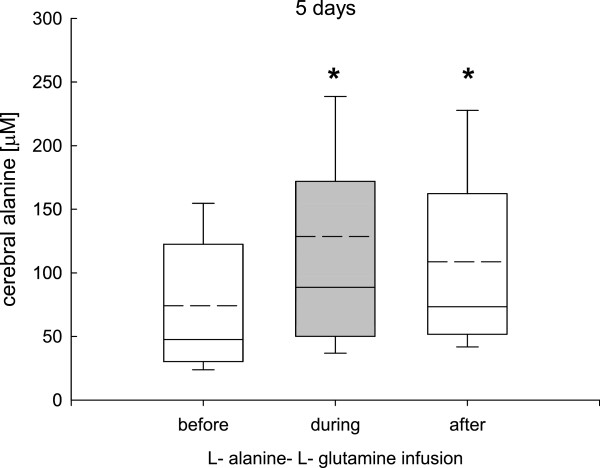
**Changes in cerebral alanine determined by cerebral microdialysis during the 5-day L-alanine-L-glutamine infusion phase.** Compared to baseline values cerebral alanine was significantly increased during the infusion phase and remained significantly elevated during the 48-hour post infusion observation phase (*P <0.001, analysis of variance, post hoc Dunn’s test).

**Figure 6:**
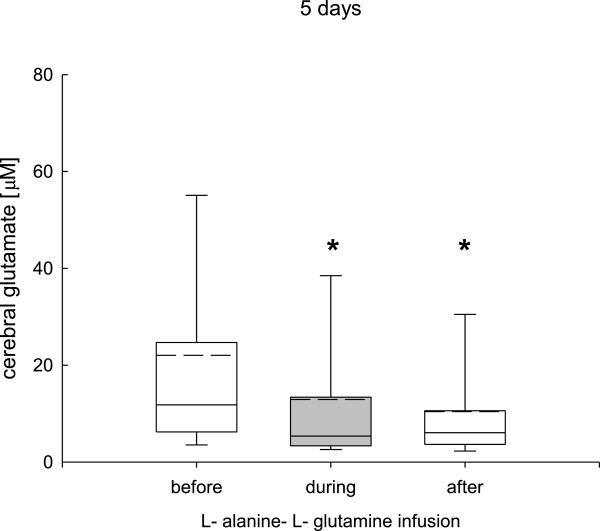
**Changes in cerebral glutamate determined by cerebral microdialysis during the 5 day L-alanine-L-glutamine infusion phase.** Compared to baseline values cerebral glutamate was significantly decreased during the infusion phase and remained significantly reduced during the 48-hour post infusion observation phase (*P <0.001, analysis of variance, post hoc Dunn’s test).

During the 24-hour infusion phase (study 1) cerebral glutamine (before infusion, 529 ± 99; during infusion, 549 ± 120; after infusion, 532 ± 110 μmol/L), alanine (before infusion, 82 ± 69; during infusion, 89 ± 90; after infusion, 77 ± 78 μmol/L), and glutamate (before infusion, 12 ± 11; during infusion, 5 ± 10; after infusion, 6 ± 9 μmol/L) remained unchanged.In study 2, prolonged L-alanine-L-glutamine infusion was associated with significantly increased cerebral glutamine after the infusion phase (Figure 4), whereas cerebral alanine was significantly increased during and after the infusion phase (Figure 5). Cerebral glutamate was significantly decreased during and after the infusion phase (Figure 6).

### Changes in cerebral glucose, lactate, lactate-to-pyruvate ratio

Extracellular cerebral glucose, lactate, and lactate-to-pyruvate ratio were not influenced by the L-alanine-L-glutamine infusion, irrespective of infusion duration (Table 1).

### Changes in neuromonitoring and sedation

During the 24-hour (study 1) and 5-day infusion phase (study 2) ICP, CPP, and ptiO_2_ remained unchanged (Table 1). Sedation was slowly reduced on days 8 and 9 after TBI, resulting in significantly increased BIS values compared to baseline values (Table 1).

### Changes in caloric requirement, respiratory quotient, laboratory values, and calculated nitrogen balance

Compared to baseline values caloric requirement (kcal/d) was significantly increased over time, reaching the highest values after the 5-day infusion phase, that is, on days 8 and 9 after TBI (Table 1). Calculated RQ remained unchanged. Hepatic and renal function, determined by changes in aspartate aminotransferase (GOT), alanine aminotransferase (GPT), and creatinine, respectively, remained normal (Table 1).

Urea and ammonia levels were significantly increased during the 5-day L-alanine-L-glutamine infusion phase, which persisted during the post-infusion observation phase (Table 1), remaining well below predefined pathologic threshold values of 15 mmol/L (urea) and 50 μmol/L (ammonia), respectively. During the 24-hour infusion phase the trend in increased urea and ammonia did not reach statistical significance (urea (median) before infusion, 2.2; during infusion, 3.3; after infusion, 2.9 mmol/L, and ammonia (median) before infusion, 12.8; during infusion, 21.7; after infusion, 18.2 μmol/L).

The calculated nitrogen balance was positive at all time points and was not changed during the 24-hour and 5-day L-alanine-L-glutamine infusion period (Table 1). The lymphocyte count was not influenced by the 24-hour or the 5-day L-alanine-L-glutamine infusion phase, respectively (Table 1).

## Discussion

Following severe TBI continuous intravenous infusion of the dipeptide L-alanine-L-glutamine at 0.5 g glutamine/kg/d and 0.25 g alanine/kg/d significantly and reversibly increased plasma glutamine and alanine concentrations. The increase in plasma glutamine was not associated with elevated plasma or cerebral glutamate or any indirect signs of secondary brain damage reflected by signs of metabolic impairment, increased ICP, decreased ptiO_2_, or sustained pharmacologic intervention to reduce ICP. Markedly elevated plasma glutamine and alanine were associated with significantly increased cerebral glutamine and alanine levels.

### Plasma glutamine and alanine in critically ill patients

Stress and inflammation as well as hormonal changes substantially influence plasma glutamine and alanine pathways to fuel energy-consuming processes [26,27]. Sustained glutamine consumption depletes the muscular glutamine storage and decreases plasma glutamine. Compared to healthy volunteers as published by Stegink *et al*., [28] and Pouw *et al*., [29] plasma glutamine was significantly decreased in the critically ill patients suffering from severe TBI, who were investigated in the current study. Hypoglutaminemia as presently shown is in line with the changes seen following, for example, elective surgery [30], neurotrauma [31,32], burn injury [33], sepsis [34], renal insufficiency [35], and chronic pulmonary disease [29]. Hypoglutaminemia, in turn, negatively influences clinical development in critically ill patients, aggravates severity of illness [1], and is associated with increased mortality and morbidity [1], thereby presenting a rationale for corrective strategies.

Contrary to the obvious hypoglutaminemia, baseline plasma alanine determined before L-alanine-L-glutamine infusion was comparable to healthy individuals [28,29] and not reduced following TBI [32]. Normal plasma alanine levels could reflect well-functioning endogenous regulatory processes or successful supply via enteral nutrition, as all patients had been successfully fed enterally before starting the L-alanine-L-glutamine infusion.

### Impact of prolonged L--alanine-L-glutamine infusion on brain parameters

Brain alanine and glutamine levels were only significantly increased if L-alanine-L-glutamine was infused for 5 days compared to 24 hours. This suggests that prolonged infusion is required to influence brain levels. The persistent increase in brain glutamine and alanine levels could either be induced by the infusion itself or by downstream alterations with reduced consumption of glutamine and alanine. Perhaps this pattern reflects time-dependent healing following TBI. However, this cannot be assessed by this study because no control group had been included.

Infusing 0.5 g glutamine/kg/d for 24 hours and 5 days did not influence measured metabolic parameters and most importantly did not increase brain glutamate levels. In addition, this infusion regimen did not induce cerebral glutamate release as arterio-jugular venous difference remained unchanged. In fact, cerebral glutamate showed a continuous significant decrease over time. Whether this decrease was induced and supported by the present infusion protocol cannot be determined as we did not include a control group. Indirect signs of glutamate-mediated cell injury were absent as reflected by unchanged cerebral glucose, lactate, lactate-to-pyruvate ratio, ICP, CPP, BIS EEG, and absent treatment escalation. This is in line with the results provided by Berg and coworkers in patients with severe head trauma (GCS ≤8) [20,21]. Contrary to the published cerebral glutamine release [21] arterio-jugular venous glutamine differences remained unchanged in the present study. This discrepancy could stem from differences in overall management, including depth of sedation, choice of sedative drugs, cooling, degree of injury, and type of brain lesions. Another possible influence could be the type of nutrition. Whereas Berg and colleagues combined the dipeptide alanine-glutamine with parenteral nutrition, we added alanine-glutamine to enteral nutrition based on the fact that enteral nutrition alone does not increase plasma glutamine comparable to its intravenous infusion. Whether the simultaneously infused amino acids in the parenteral nutrition additionally and differentially influences brain metabolism, and specifically glutamine-mediated changes, remains to be investigated in a separate study.

The present investigations extend the available knowledge as we investigated a higher dose, that is, 0.5 g glutamine/kg versus 0.34 g/kg and a longer duration, that is, 24 hours and 5 days versus 20 hours [20,21]. The obtained data show that intravenous infusion of glutamine at a dose higher than currently recommended (0.5 versus 0.34 g/kg/d) does not increase cerebral glutamate or induce glutamate-mediated cerebral injury. Thus, this dose and the longer duration appear to be safe in patients with severe TBI.

Infusing a higher dose of alanine, that is, 0.25 g alanine/kg/d for 24 hours and 5 days compared to 0.17 g alanine/kg/d for 20 hours [20,21] significantly increased cerebral alanine levels with unchanged arterio-jugular venous difference. Whether the significant and persisting increase in cerebral alanine contributed to the significantly decreased cerebral glutamate by fueling gluconeogenesis, thereby sparing glutamate and glutamine as energy-delivering compounds [36,37] or influencing ammonia and lactate transfer [38] cannot be answered by the present study. Increased cerebral alanine levels were not associated with a decrease in BIS EEG values or reduction in administered midazolam dose, which questions its role in neuronal inhibition under the conditions of the present study design [39].

### Which glutamine and alanine dose and which duration of infusion could be optimal?

Contrary to previous investigations, a higher glutamine and alanine dose was infused in the current study, that is, 0.75 g versus 0.5 g L-alanine-L-glutamine/kg/d with the aim of normalizing and maintaining normalized plasma glutamine levels. Albeit a significant increase in plasma glutamine even this higher dose, that is, 0.5 g versus 0.35 g glutamine/kg/d failed to completely normalize plasma glutamine levels in all patients. This strongly suggests a sustained requirement. Whether this can be met by infusing an even higher glutamine dose in these patients is currently unclear. As shown in non-TBI patients glutamine must be infused at 0.57 or 0.86 g/kg/d to successfully normalize plasma glutamine levels [40].

Plasma alanine was also significantly increased by the currently investigated protocol. Baseline plasma alanine values had already been normal. Thus, the necessity to further increase plasma alanine seems questionable. However, the present study does not allow us to determine potential positive intracellular effects in terms of alanine-mediated sustained protein synthesis and decreased protein degradation to define required plasma alanine levels.

Duration of L-glutamine-L-alanine infusion determines persistence of elevated plasma levels. In this context, plasma glutamine returned to baseline values within 30 minutes or 8 hours following a 30-minute versus a 4-hour infusion, respectively [41,42]. This might explain the significantly increased plasma glutamine and alanine levels during the 5-day versus the 24-hour infusion period observed in the present study. As reflected by the significantly decreased plasma glutamine and alanine levels during the post-infusion period, glutamine and alanine did not accumulate. This is in line with previous reports [43].

Clinical investigations with a larger number of patients are needed to define the optimal dose and duration. The present investigation was not designed to assess the impact of L-alanine-L-glutamine infusion on clinical outcome in this specific population of critically ill TBI patients. The present results could aid in defining the required dose and duration in future clinical trials. The results and the glutamine dose used in the recently published large-scale clinical trials do not provide clear answers as to which glutamine dose is to be used. In the SIGNET trial [15], glutamine is discussed as most likely having been too low. In the REDOXS study, the total glutamine dose from simultaneous enteral and intravenous administration might have been too high in patients suffering from multi-organ failure with unresolved shock, renal failure, and insufficient nutrition [44].

### Side effects of prolonged infusion of a higher glutamine and alanine dose

In line with amino acid-induced ammoniagenesis and ureagenesis [45-48] the presently investigated patients showed increased ammonia and urea levels during L-alanine-L-glutamine infusion compared to baseline values, as they received an average total nitrogen load of 19.7 g/d (median enteral nitrogen load 9.7 g/d, median parenteral nitrogen load from L-alanine-L-glutamine infusion 10 g/d). The elevated urea and ammonia levels did not reach worrisome levels and were not associated with signs of neurologic and metabolic deterioration as ICP remained unchanged, and glutamate was significantly decreased in the face of stable brain metabolism reflected by unchanged cerebral lactate and lactate-to-pyruvate ratio. Apparently, critically ill patients, even with normal hepatic function according to laboratory values and medical history, respond differently to protein load compared to healthy individuals who can tolerate a glutamine load of 0.65 g glutamine/kg/d without increased plasma ammonia levels [49]. As the increased blood ammonia and urea levels were well within the normal range this metabolic response is not considered a safety concern. The currently investigated patients did not show any signs of hepatic or renal dysfunction. Thus, the investigated dose and duration cannot be extrapolated to all patients. The primary aim of these investigations was to determine pharmacodynamic changes. In this context, the missing control group does not allow us to differentiate influences on ammoniagenesis and ureagenesis attributable to nutrition or alanyl-glutamine infusion.

### Limitations of the present study

The low number of patients and the chosen study design without having included a control group does not allow us to recommend the investigated dose and duration in all patients. The presently investigated patients comprise a well-defined subpopulation, which does not allow us to generalize as these patients did not suffer from any additional organ dysfunction apart from brain injury, did not require dialysis or hemofiltration, and had a well-functioning gastrointestinal tract receiving enteral nutrition according to the individually determined caloric requirement.

## Conclusions

Low plasma glutamine levels were significantly and reversibly increased by continuous intravenous infusion of 0.75 g L-alanine-L-glutamine, reaching highest plasma glutamine and alanine levels during the 5-day infusion phase. Infusion of L-alanine-L-glutamine for 5 days was required to significantly increase brain glutamine and alanine levels compared to the 24-hour infusion phase. The absent increase in glutamate as well as absent indirect signs of glutamate-mediated cerebral injury underscore the reported safety of 1) infusing L-alanine-L-glutamine and 2) using the investigated dose in patients with severe TBI. To normalize plasma glutamine levels higher glutamine amounts exceeding 0.5 g/kg/d might be required.

## Key messages

• Continuous intravenous infusion of L-alanine-L-glutamine at 0.75 g/kg/d for 5 days significantly increased plasma as well as cerebral glutamine and alanine levels

• This increase in plasma glutamine was not associated with elevated plasma and cerebral glutamate, and cerebral glutamate release.

• The continuous infusion of L-alanine-L-glutamine at 0.75/g/kg/d for 24 hours significantly increased plasma glutamine and alanine levels without influencing cerebral glutamine and alanine levels.

• Overall, continuous intravenous infusion of a higher L-alanine-L-glutamine dose (0.75 g/kg/d) for up to 5 days was not associated with signs of glutamate-mediated cerebral injury.

## Abbreviations

BIS EEG: bispectral index electroencephalogram; CPP: cerebral perfusion pressure; CT: computed tomography; GCS: Glasgow coma scale; GOT: aspartate aminotransferase; GPT: alanine aminotransferase; HPCL: high performance liquid chromatography; ICP: intracranial pressure; kcal: Kilocalories; ptiO_2_: brain tissue oxygen; RQ: respiratory quotient; SjvO_2_: jugular venous oxygen saturation; TBI: traumatic brain injury.

## Competing interests

JFS has received a speaker’s honorarium from Fresenius Kabi. Following study completion JFS joined Fresenius Kabi in permanent employment. The remaining authors declare that they have no competing interests.

## Authors’ contributions

All authors have contributed substantially to the work: conception (JFS), data acquisition (MN, MF, JS, GB), analysis (AF); all authors contributed to drafting the work and revising it critically for important intellectual content; all authors approved of the final version to be published.
